# Aberrant Spontaneous Brain Activity in Coronary Heart Disease Using Fractional Amplitude of Low-Frequency Fluctuations: A Preliminary Resting-State Functional MRI Study

**DOI:** 10.1155/2022/2501886

**Published:** 2022-06-03

**Authors:** Simin Lin, Yi Han, Shaoyin Duan, PuYeh Wu, Naiming Wu, Ting Xie, Qin Li, Qing Lu, Hengyu Zhao

**Affiliations:** ^1^Department of Radiology, Xiamen Cardiovascular Hospital of Xiamen University, School of Medicine, Xiamen University, Xiamen 361006, China; ^2^Fujian Provincial Key Laboratory of Ophthalmology and Visual Science, Eye Institute of Xiamen University, School of Medicine, Xiamen University, Xiamen 361100, China; ^3^Department of Radiology, Zhongshan Hospital of Xiamen University, School of Medicine, Xiamen University, Xiamen 361001, China; ^4^GE Healthcare, Beijing 100020, China

## Abstract

**Objective:**

This study is aimed at exploring the spontaneous brain activity changes by measuring the fractional amplitude of low-frequency fluctuations (fALFF) and their relationship with clinical characteristics in patients with coronary heart disease (CHD).

**Methods:**

Coronary heart disease patients (*n* = 25) and age, gender, and education level-matched control subjects (controls, *n* = 35) were included. The grey matter volume (GMV) and fALFF values were calculated to assess the difference in brain structure and function between the two groups, respectively. Correlation analyses between the fALFF values and clinical characteristics were further assessed in CHD patients. In addition, receiver operating characteristic (ROC) curves were conducted to access the diagnostic ability of the fALFF method.

**Results:**

There was no significant difference in GMV between the CHD and control groups. Compared with the control group, patients with CHD showed significantly decreased fALFF in the left precentral/postcentral gyrus and increased fALFF in the right inferior cerebellum. Patients with a history of myocardial infarction (MI) showed significantly decreased fALFF values of the right inferior cerebellum than patients without MI. There was no significant correlation between the fALFF values in specific brain regions and disease duration. Furthermore, the ROC curves of abnormal brain regions showed the perfect accuracy of the fALFF value in distinguishing between CHD patients and controls.

**Conclusion:**

CHD demonstrated aberrant neural activity in specific brain regions mainly related to sensorimotor networks and pain processing, which may contribute to understanding the underlying neurological mechanism of CHD.

## 1. Introduction

Coronary heart disease (CHD), one of the primary causes of death, poses serious threats to physical health [1]. Angina pectoris is the most common symptom of CHD. Over the past decade, due to timely and effective clinical interventions, the mortality rate of CHD declined by 14.6% in the United States [2]. Although CHD survival rates have improved in recent years, it is an urgent requirement to cope with the effects of CHD “successful aging.” Increasing cases of CHD-related brain function decline were reported in recent years [3, 4]. van de Vorst et al. indicated that people with CHD had an increased risk of cognitive decline and higher rates of depression and anxiety compared with normal controls [5]. Hence, recent research focus turned to the brain function disorder caused by CHD. Neuroimaging has proved to be an effective tool for assessing brain function. Many previous MRI studies demonstrated that patients with CHD have white matter lesion, grey matter atrophy, and cerebral blood flow change [6–10]. Nevertheless, little is known about the changes of CHD in brain activity. Measurement of neural activity is a suitable method for researching the potential impacts of CHD on brain function [10].

Resting-state functional MRI (rs-fMRI), a useful and noninvasive method for neural activity assessment, has attracted substantial attention in recent years [11]. Resting-state fMRI detects spontaneous neural activity independent of stimulation or task design constraints via detecting blood oxygen level-dependent (BOLD) signals [12, 13]. In the field of cardiovascular, Bernard et al. used resting-state fMRI to study alterations of functional connectivity in acute coronary syndrome and showed evidence of abnormal neural networks [14]. This research only concentrated on the alteration of functional connectivity between two aberrant brain areas. Based on previous reports [4, 15], we speculate that the influence of CHD on the brain might be global; thus, it is of certain significance to analyze the whole brain activity in patients with CHD.

The fractional amplitude of low-frequency fluctuations (fALFF) is regarded as a novel method to assess global spontaneous brain activity. The fALFF is used to measure the amplitude of a spontaneous, low-frequency (0.01~0.08 Hz) BOLD signal, which reflects the aberrant brain activity at baseline and offers valuable information about brain activity [16]. In contrast with the amplitude of low-frequency fluctuations (ALFF), fALFF can effectively suppress physiological noise, decrease interference with cerebrospinal fluid, and show high sensitivity to detect spontaneous neural activity [16]. In recent years, ALFF and fALFF analyses have been widely applied in a series of neuropsychiatric disorders and have been used in the research on pain mechanisms, showing abnormalities of neural activity in pain-related diseases such as Crohn's disease, irritable bowel syndrome, and chronic low back pain [17–19].

In this study, we mainly explored the alterations in spontaneous neural activity by measuring fALFF in patients with CHD and their relationship with clinical features. We hypothesized that (1) patients might have abnormal fALFF values in specific brain areas and (2) the alterations of spontaneous neural activity may provide new insight into the neurological mechanism of CHD.

## 2. Materials and Methods

### 2.1. Subjects

The experiment was authorized by the Ethics Committee of Xiamen Cardiovascular Hospital of Xiamen University. All participants in the current research obtained written informed consent. Twenty-six patients with CHD were enrolled from the Xiamen Cardiovascular Hospital of Xiamen University from May 2020 to May 2021. Thirty-six volunteers matched in age, gender, and education level were enrolled from the local community as control subjects (controls).

According to the current guidelines, the diagnostic criteria for CHD are either provocable myocardial ischemia or at least one coronary artery stenosis of 50% or greater [20, 21]. Inclusion criteria for CHD patients were (1) duration of disease of at least 7 days, (2) left ventricular ejection fraction (LVEF) greater than 50%, and (3) age range between 40 and 70. To reduce the insidious effects of neurodegeneration on cognitive status, elderly patients older than 70 years were not recruited. Inclusion criteria for control subjects were (1) no symptoms or biochemical evidence of cardiovascular disease and (2) no obvious abnormalities in transthoracic echocardiography and ECG. Exclusion criteria for all participants were (1) history of brain lesions and neurological or psychiatric disorders, (2) aphasia and severe visual or hearing deficiency, (3) cardiomyopathy/severe or complex arrhythmias/congestive heart failure, (4) history of trauma or surgery in the last three months (excluding percutaneous coronary intervention), (5) history of alcohol and toxicology abuse, (6) left-hander, and (7) MRI contraindications.

### 2.2. MRI Parameters

All MRI data were obtained using a 3-Tesla MRI scanner (SIGNA Pioneer, GE Healthcare, Milwaukee, WI, USA) at the Xiamen Cardiovascular Hospital of Xiamen University. All participants were reminded to close their eyes but stay awake and keep silent during the whole scanning. We used earplugs to diminish the acoustic noise and used foam pads to minimize motion-related artifacts during scanning.

High-resolution structural images were acquired using a 3D T1-weighted inversion recovery prepared fast spoiled gradient-recalled echo (IR FSPGR) pulse sequence. The parameters of structural images are as follows: echo time (TE) = 3.2 ms, repetition time (TR) = 8.3 ms, inversion time (TI) = 450 ms, flip angle (FA) = 12°, field of view (FOV) = 240 mm × 240 mm, thickness = 1.0 mm, matrix = 240 × 240, gap = 0 mm, voxel size = 1.0 mm × 1.0 mm × 1.0 mm, and number of slices = 168 or 184.

Resting-state fMRI images, including 185 volumes, were acquired by a 2D gradient-recalled echo echo-planar imaging (GRE-EPI) pulse sequence in the axial plane. The parameters of functional images are as follows: TE = 30 ms, TR = 2000 ms, FA = 90°, FOV = 240 mm × 240 mm, thickness = 4.0 mm, matrix = 64 × 64, gap = 0 mm, voxel size = 3.75 mm × 3.75 mm × 4.0 mm, and number of slices = 36.

### 2.3. Voxel-Based Morphometry Analysis

The 3D T1-weighted images were analyzed using the VBM8 toolbox (http://dbm.neuro.uni-jena.de/vbm) based on SPM8 (http://www.fil.ion.ucl.ac.uk./spm/). First of all, all structural images were visually screened to exclude poor-quality images and subsequently segmented into white matter (WM), grey matter (GM), and cerebrospinal fluid (CSF) with default parameters by the standard segmentation model. After an affine-registered map of GM concentrations to Montreal Neurological Institute (MNI) space, the images of GM concentrations were nonlinear deformations using the DARTEL algorithm and then resampled to 1.5 mm^3^ isotropic voxel size. The normalized and modulated tissue probability map of GM volume was acquired by multiplying the GM concentration map by the nonlinear determinant. Finally, the images of GM were smoothed with a full-width at half-maximum (FWHM) of 8 mm.

### 2.4. Functional Data Preprocessing and Analysis

Firstly, all images were examined by MRIcro (https://www.MRIcro.com), and images with severe artifacts were removed. Then, the fMRI data were preprocessed and analyzed by CONN (https://www.nitrc.org/projects/conn) implemented in MATLAB 2013b (https://www.mathworks.com/products/matlab). Specifically, the data were preprocessed by the following procedures: (1) removal of the initial time point: the initial five scan volumes were removed for each participant to reach a signal equilibrium and reduce the deviation; (2) slicing timing correction: the collected layers of each scan volume were calibrated to a correct time point; (3) motion correction: all scan volumes were realigned to correct head movements, and participants with head motion more than 2.5 mm of maximal translation or rotation more than 2.5° were excluded; (4) normalization: all functional images were spatially standardized to the MNI standard space after registration with the structural images to minimize differences between individuals and resampled to 3 mm voxels; (5) smoothing: all functional images were smoothed with a FWHM of 6 mm. The purposes of smoothing were (a) to make the data follow a Gaussian distribution, (b) to increase the signal-to-noise ratio, and (c) to compensate for the registration error in the spatial normalization step; (6) linear detrending; and (7) linear regression of confounding effects: realignment (including six head motion parameters and six head motion parameters one time point before), scrubbing, average BOLD signal of WM, and CSF were regressed out.

After preprocessing, the time series were transformed to the frequency domain utilizing fast Fourier transform (FFT). Subsequently, the square root of the power spectrum was calculated, and then, the averaged square root was acquired across 0.01-0.08 Hz to obtain the ALFF. fALFF was subsequently calculated by dividing the power spectrum of the low-frequency range of 0.01-0.08 Hz by the power spectrum of the entire frequency range of 0-0.25 Hz [16]. Finally, to minimize the global influence, the fALFF values were divided by the global mean fALFF values for standardization, which was taken as the mean fALFF.

### 2.5. Statistical Analysis

Differences in demographics and clinical characteristics between CHD and control groups were calculated using SPSS 26.0. The two-sample *t*-test was utilized for continuous variables, and the chi-squared test was used for proportions. The significance threshold of the statistical test was 0.05.

Regarding the fMRI data, differences in GVM and fALFF values of each voxel between CHD and control groups were compared by an independent two-sample *t*-test using SPM8 software implemented in MATLAB. Age, gender, and education levels were imported as covariates to eliminate the impact of confounding covariates on fALFF values. In the meantime, cardiovascular risks (hypertension, diabetes, and smoking) can affect brain structure and function and further interfere with the reliability of the results [10, 22, 23]. Therefore, hypertension, diabetes, and smoking were matched in the CHD and control groups to reduce potential confounding impacts. The cluster-level family-wise error (FWE) method was applied for multiple comparison correction, and a cluster-defined threshold was set to 0.001, and a corrected cluster significance was *p* < 0.05.

The course of disease does not conform to the normal distribution, so the Spearman correlation was applied to identify the association between the mean fALFF in altered brain areas and disease duration. The independent two-sample *t*-test was applied to analyze the association between the fALFF and the history of myocardial infarction (MI). The statistical threshold was set at *p* < 0.05. Additionally, we utilized the receiver operating characteristic (ROC) curve analysis to access the diagnostic ability of the fALFF method in altered brain regions in distinguishing CHD patients from controls. The area under the curve [24] was calculated to represent the degree of accuracy. Accuracy was considered perfect when the AUC was more than 0.9.

## 3. Results

### 3.1. Demographics and Clinical Characteristics

A total of 62 participants (26 patients and 36 control subjects) were recruited for the current study, and one patient and one control were eliminated due to excessive movement of the head. Sixty subjects (25 patients and 35 control subjects) were included for the following analysis, and no significant differences were observed in gender (*p* = 0.757), age (*p* = 0.064), years of education (*p* = 0.513), BMI (*p* = 0.942), history of smoking (*p* = 0.775), hypertension (*p* = 0.895), diabetes (*p* = 0.898), and LVEF (*p* = 0.784) between the CHD patients and control subjects. Details are summarized in Table 1.

### 3.2. Comparison of GMV

For the VBM analysis, no statistical difference was observed in GMV between the CHD and control groups (*p* > 0.05, cluster-level FWE-corrected). Overall, however, the GMV of patients with CHD was lower than that of the control group. Details about total grey and white matter volume are shown in Table 2.

### 3.3. Differences in fALFF

**Figure 1:**
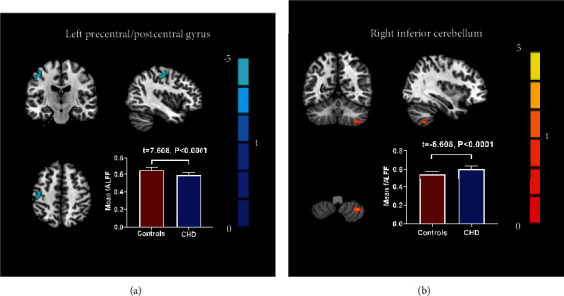
Mean fALFF differences between the control subjects and CHD patients. Compared to control subjects, (a) shows decreased fALFF values of the left precentral/postcentral gyrus in CHD patients. (b) Shows increased fALFF values of the right inferior cerebellum in CHD patients. Error bars indicate the standard deviation. fALFF = fractional amplitude of low-frequency fluctuations; CHD = coronary heart disease.

Compared with the control group, patients with CHD showed decreased fALFF values in the left precentral/postcentral gyrus and increased fALFF values in the right inferior cerebellum (Table 3 and Figure 1).

### 3.4. Correlation Analysis between fALFF and Clinical Characteristics

**Figure 2:**
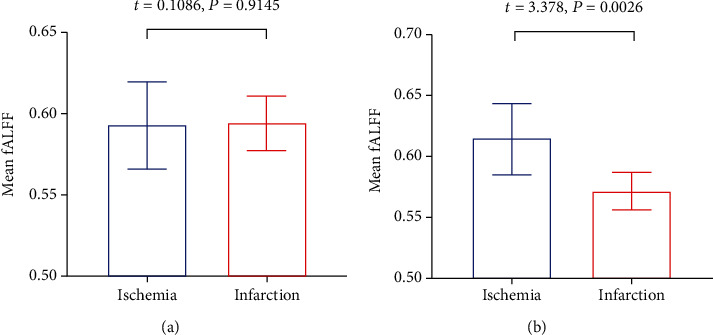
Mean fALFF differences between the ischemia and infarction groups. (a) Shows fALFF values in the left precentral/postcentral gyrus. (b) Shows fALFF values in the right inferior cerebellum.

Patients of CHD were divided into the ischemic group and the infarction group, without a history of myocardial infarction (MI) and with a history of MI, respectively. The fALFF values of the right inferior cerebellum were significantly lower in the infarction group (*p* = 0.0026). No statistical difference was discovered in fALFF values of the left precentral/postcentral gyrus between the ischemic and infarction groups (*p* = 0.9145) (Figure 2). There was also no significant correlation between the aberrant fALFF values and disease duration (*p* > 0.05).

### 3.5. Receiver Operating Characteristic Curve

**Figure 3:**
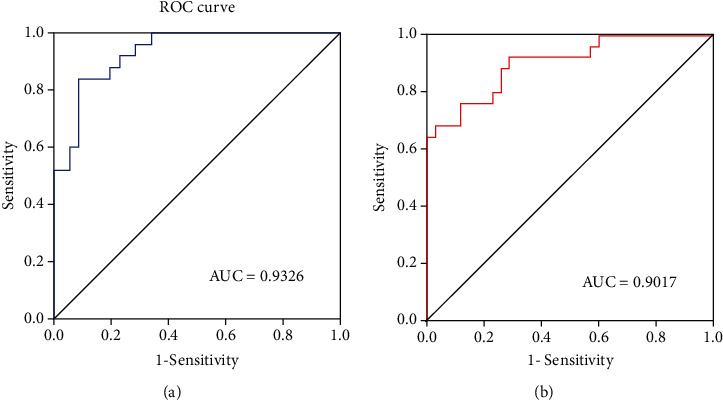
ROC curve analysis of fALFF values for altered brain regions. (a) Left precentral/postcentral gyrus. AUC were 0.9326 (*p* < 0.0001; 95% CI: 0.8740-0.9911); (b) right inferior cerebellum. AUC were 0.9017 (*p* < 0.0001; 95% CI: 0.8233-0.9801). ROC = receiver operating characteristic; AUC = area under the curve; CI = confidence interval.

We speculated that the significant differences in fALFF values might be helpful imaging biomarkers to differentiate CHD patients from controls; thus, we further performed ROC curve analysis with the averaged fALFF values in these altered brain regions. The AUC results of fALFF values in the left precentral/postcentral gyrus and right inferior cerebellum were 0.9326 and 0.9017, respectively, indicating the accuracy was perfect (Figure 3).

## 4. Discussion

We designed this study to determine whether patients with CHD display abnormal brain activity compared with the control group. Coronary heart disease is an urgent, rapidly developing critical disease with high disability and mortality, which seriously endangers human health [1, 25]. Previous studies have indicated that patients with CHD exhibited an increased risk of mild cognitive and emotional dysfunction [4, 5]. Therefore, our study is aimed at preliminarily exploring the underlying neural mechanisms by measuring spontaneous brain activity changes of CHD via resting-state fMRI. To the best of our knowledge, it is the first time to investigate the spontaneous brain activity alterations in patients with CHD.

The present results demonstrated no significant difference was discovered in GMV between the two groups and revealed the aberrant neural activity in CHD patients by measuring fALFF using rs-fMRI. The fALFF reflects the spontaneous neural activity by measuring changes in the BOLD signal. The fALFF can effectively suppress physiological noise and improve the specificity and sensitivity of spontaneous neuronal activity compared with ALFF [16]. Compared with controls, the CHD patients showed lower fALFF values in the left precentral/postcentral gyrus and higher fALFF values in the right inferior cerebellum. Compared with patients without a history of MI, MI patients showed lower fALFF values in the right inferior cerebellum. However, there was no statistical correlation between the fALFF values in brain areas and the course of the disease. In addition, no significant difference was discovered in GMV between the two groups.

Our current research demonstrated no evidence of a statistical difference in GMV between patients with CHD and matched controls, which is inconsistent with previous studies. Koschack and Irle demonstrated smaller GMV in the hippocampus, which may be a biomarker to predict later cognitive decline or dementia [26]. Almeida et al. revealed that CHD was related to grey matter atrophy in a series of brain regions, including the precentral and postcentral cortex, the left medial frontal lobe, and right temporal lobe, some of which were of great significance in cognitive function and behavior [27]. A case-control study found that the cerebral cortex volume of the left pars triangularis, left fusiform, and left superior temporal gyrus decreased significantly in patients with CHD combined with cognitive impairment [8]. GMV abnormalities in the brain regions were also inconsistent in the above studies. Shorter exposure time to vascular risk factors in relatively young CHD patients in this study may be contributing to explaining these results. Different inclusion criteria between the CHD and control groups are also one of the reasons that account for the inconsistent results. Other potential reasons include differences in structural MRI data processing methods (VBM vs. CURRUY) and statistical analysis approaches.

Previous neuroimaging reports showed the atrophy of grey matter and reduction of cerebral blood flow in precentral and postcentral gyri in patients with CHD [6, 9]. The current study showed lower fALFF values in the left precentral/postcentral gyrus in patients with CHD. Therefore, we speculated that the precentral and postcentral gyri may be the vital brain regions closely associated with CHD. It is well known that the postcentral gyrus is the primary somatosensory cortex (S1) and the precentral gyrus is the primary motor cortex (M1), which play important roles in primary sensory and motor control functions, respectively. The decreased neural activity of the postcentral and precentral gyri, the crucial components of the sensorimotor network, is associated with the modulation dysfunction of the sensorimotor network [28, 29]. Both postcentral and precentral gyri are involved in pain processing, which is a multidimensional experience involving sensory discrimination, cognition evaluation, and affective motivation [30]. A large number of researches have proven the essential roles of the postcentral and precentral gyri in chronic pain-related [17, 18, 31]. Therefore, we hold the opinion that the decreased neural activity in the postcentral and precentral gyri may be associated with angina pectoris. The postcentral gyrus is a component of the homeostatic-afferent network that participates in the central processing of visceral pain [32] and plays a vital role in the discrimination and localization of pain [33]. Kong et al. reported that S1 was involved in the pathological pain state of chronic low back pain [34]. Liu et al. demonstrated that regional cerebral blood flow in S1 was highly correlated with pain intensity in patients with postherpetic neuralgia [35]. Some neuroimaging studies had confirmed that chronic pain patients were also associated with abnormal excitability in the cortical motor area [19, 36]. Motor cortex stimulation can relieve plenty of chronic pain syndromes [37]. Bernard et al. suggested that the precentral gyrus may be a vital brain area as a target for chronic pain treatment in somatoform pain disorders [14]. Meanwhile, the precentral gyrus is located in the frontal lobe, which is associated with the regulation of emotion and plays an important part in emotional memory storage [17]. Zhang et al. indicated that decreased volume of grey matter in the precentral gyrus may be associated with an increased risk of depressive disorder [38]. Xiong et al. pointed out that the altered cortical thickness of the left precentral gyrus may stand for state-independent abnormalities in major depressive disorder [39]. Plenty of studies demonstrated that patients with CHD have an increased incidence of depression, but the exact neural mechanism is unclear [40, 41]. The decreased fALFF in the precentral gyrus may contribute to explaining the underlying neural mechanism of increased risk of depression in CHD. Previous neuroimaging studies also demonstrated that the motor cortex may be involved in cognitive functions, including implicit motor learning ability [42] and working memory [43]. Bush et al. demonstrated that the precentral gyrus participates in the executive control of response inhibition [24]. Many neuropsychological tests have shown that CHD is associated with an increased risk for cognitive decline, especially executive function, which we suspect may be associated with lower neural activity in the precentral gyrus [14]. These pieces of evidence from neuroimaging research may indicate that the precentral gyrus is not only involved in motor control but also in pain modulation and emotional and cognitive processing. The significantly lower fALFF values in the left precentral/postcentral gyrus in patients with CHD may be involved in the dysfunction of the sensorimotor network and pain coding and even associated with emotional and cognitive processing.

Compared with the control group, the CHD patients exhibited higher fALFF values in the right inferior cerebellum. The cerebellum is regarded as a major motor control region [44]. Several studies have proved that the cerebellum also participates in the processing of emotion and cognition [29, 45]. The low ReHo value of the cerebellum in patients with major depressive disorder suggested that the cerebellum was involved in mood regulation [46]. The cerebellum has recently been proven to play a part in somatosensory processing, including nociception [47]. Moulton et al. confirmed that the cerebellum participated in pain processing [30]. We supposed that the increased brain activity in the right cerebellum may have part of compensatory effects with regard to both motor and nonmotor dysfunctions due to brain activity reduction in the precentral and postcentral gyri. We indicated that the fALFF values of the right inferior cerebellum in patients with MI were lower than those in patients without MI, which may indicate that the compensatory effects of MI patients are lower than that of patients without MI. Therefore, patients with MI may show a greater possibility of brain function decline.

We acknowledged that several limitations still exist. First, the deficiency of objective psychological or cognitive evaluation restricted our interpretation of the outcomes, and the exact mechanism of altered neural fALFF activity in CHD remains unclear. Second, the sample size was relatively small, especially the number of patients with a history of MI, which could limit the statistical capacity to detect tiny changes in the brain. Third, part of patients with CHD was receiving multiple drugs that might have possible impacts on brain activity. Medication-naive groups should be considered to exclude this potential bias in further research.

## 5. Conclusions

Our investigation demonstrated that there were significant changes in spontaneous brain activity prior to structural changes in patients with CHD. These abnormal brain activities were mainly located in the left precentral/postcentral gyrus and the right inferior cerebellum. Spontaneous brain activity abnormalities may provide useful hints regarding underlying neural mechanisms of abnormal brain function in CHD.

## Figures and Tables

**Table 1:** Demographic and clinical characteristics.

	Control subjects (*n* = 35)	CHD patients (*n* = 25)	Statistics	*p* value
Demographic characteristics				
Gender: men (%)	21 (60%)	14 (56%)	*χ* ^2^ = 0.096	0.757^b^
Age (year)	53.74 ± 7.66	57.44 ± 7.18	*t* = −1.892	0.064^a^
Education (year)	10.03 ± 3.51	9.32 ± 4.77	*t* = 0.659	0.513^a^
BMI (kg/m^2^)	23.66 ± 2.70	23.71 ± 2.62	*t* = −0.073	0.942^a^
Clinical characteristics				
Hypertension, *n* (%)	19 (54%)	14 (56%)	*χ* ^2^ = 0.017	0.895^b^
Diabetes, *n* (%)	4 (11%)	4 (16%)	*χ* ^2^ = 0.016	0.898^b^
Smoking, *n* (%)	11 (31%)	7 (28%)	*χ* ^2^ = 0.082	0.775^b^
LVEF (%)	65.46 ± 6.80	65.96 ± 5.93	*t* = −0.276	0.784^a^
Disease duration (month)	N/A	37.99 ± 43.13	N/A	N/A
History of MI, *n* (%)	N/A	6 (24%)	N/A	N/A

The data are shown as the mean values ± standard deviations. N/A = not applicable; BMI = body mass index; LVEF = left ventricular ejection fraction; MI = myocardial infarction. ^a^The *p* value was obtained by a two-sample *t*-test. ^b^The *p* value was obtained by the chi-squared test.

**Table 2:** Total brain volume between control subjects and CHD patients.

	Control subjects (*n* = 35)	CHD patients (*n* = 25)	Statistics	*p* value
Total grey matter volume	634.47 ± 51.99	621.33 ± 47.07	*t* = 1.003	0.320^a^
Total white matter volume	533.36 ± 69.60	515.40 ± 49.30	*t* = 1.106	0.273^a^

The data are shown as the mean values ± standard deviations. ^a^The *p* value was obtained by a two-sample *t*-test.

**Table 3:** Differences in fALFF values between control subjects and CHD patients.

Brain regions	Peak MNI coordinates			Cluster size	*t* value
	*x*	*y*	*z*		
CHD < controls					
Left precentral/postcentral gyrus	-39	-15	60	83	4.4716
CHD > controls					
Right inferior cerebellum	30	-51	-57	25	-5.2498

^∗^ *x*, *y*, and *z* are the locations of the peak voxels in standard MNI coordinates. FWE correction, cluster-level: *p* < 0.05. MNI = Montreal Neurological Institute.

## Data Availability

The data used to support the findings of this study are available from the corresponding author upon request.
